# Surgery

**Published:** 1896-02

**Authors:** John Parmenter

**Affiliations:** Buffalo, N. Y., Professor of anatomy and adjunct professor of clinical surgery, Medical Department University of Buffalo


### Progress in Medical Science.

### SURGERY.

Conducted by JOHN PARMENTER, M. D., Buffalo, N. Y.,
Professor of anatomy and adjunct professor of clinical surgery, Medical Department University of Buffalo.

### PRURITUS ANI.

ADLER, LEWIS H., Jr., (*Philadelphia Polyclinic*,) well summarises an article on the Etiology and symptomatology of pruritus ani, as follows :

*Etiology.*—The causes of pruritus ani are both local and general. There are many cases affected with this disease, however, in which we are unable to discover any assignable reason for its existence, and it may then be considered, as stated by Mr. Allingham, as a pure neurosis, being occasioned or greatly aggravated by mental worry or overwork.

*Local Causes.*—The leucorrheal discharges of women often excite the disease by remaining in contact with the skin of the perineum and developing an eczema. In children especially the disease may result from the presence of oxyuris vermicularis in the rectum. Peduculi or scabies, or the presence of a vegetable parasite, may occasion a pruritus. Improper diet and highly seasoned foods may excite the disease. Hemorrhoids, polypoid growths, fissure or fistula, from the irritation they set up and the abnormal secretion they produce, may occasion pruritus ; likewise chronic diarrhea or dysentery may induce the disease. Erythema, herpes and any variety of eczema, whether acute or chronic, may give rise to this affection. Stricture and inflammation of the upper portion of the urethra sometimes occasions itching about the anus. Pruritus frequently depends upon a varicose condition of the veins of the rectum, just as occurs in a similar condition of the veins of the leg. The itching may be due to uterine disorder. In some cases the disease is due to uncleanliness and insufficient ablution of the anus ; and, finally, the use of hard or printed substances for detergent purposes may excite a pruritus ani.

*General Causes.*—Gouty subjects and persons with a more or less marked lithic acid diathesis are predisposed to attacks of pruritus ani. A not infrequent cause of pruritus is derangement of the liver, which may or may not be associated with constipation. Diabetes will often give rise to this disease, as likewise may chronic constipation. Excessive smoking may produce this disorder. The free indulgence in alcoholic liquids, or of coffee, may induce this disease. Excesses at the table, combined with a lack of proper exercise, not only predispose the individual to pruritus, but also may become the exciting cause of the malady. Pruritus has also been ascribed to diseases of the spinal cord and brain.

*Symptomatology.*—The most prominent and, indeed, the only essential symptom of this disease, is the severe itching. In the majority of cases the irritation is worse at night, especially when the patient gets warm in bed. The intolerable itching renders sleep almost impossible, and when the sufferer does fall off into a fitful slumber he frequently awakes himself by scratching. The itching is so intense that it is impossible to avoid scratching, which, instead of giving relief, only adds to the trouble.

Nervous and excitable persons are prone to the attacks of pruritus, during the day as well as night; especially is this the case after exercise or on leaving the cold air and coming into a warm room.

In marked cases of pruritus a characteristic condition of the disease is the loss of the natural pigment of the part, and the skin is not supple, but has a peculiarly harsh and rough feel.

---

### A NEW STYPTIC.

ROSWELL PARK (*Medical News*, November 16, 1895,) reviews his experiences with a spray of 5 per cent. solution of antipyrin, made up with sterilised water, as a styptic in surgical operations. He has found it an especially useful measure in parenchymatous oozing which complicated operations. It has been tried by many surgeons and found useful, having no deleterious effects, no matter where it was used. He has since found that a combination of antipyrin and tannic acid is still more useful. This mixture precipitates a thick, gummy cohesive substance, which offers the most ideal styptic for certain purposes. An alcoholic solution of tannic acid is used and antipyrin added in quantity sufficient to form a precipitate of required consistency. This substance is particularly useful in hemorrhage from bone, for instance, in operations upon the cranium. A small piece of sponge or cotton sopped in it may be forced into a bleeding tooth socket, and in many other ways it may be very useful. There is but one attendant difficulty, due to its remarkable cohesiveness, that when the time comes for detachment or separation it is difficult to remove it. Sometimes it has been necessary to wait for the formation of granulations and separation by natural process.

---

ROSS W. MARTIN (*International Journal of Surgery*, December, 1895,) has a paper on the application of plaster-Paris and its effects, principally in cases of tubercular ostitis, hip-joint diseases and osteomyelitis. He prefers in most cases the plaster-Paris dressing to other mechanical appliances, principally because it can be made to fit more accurately, and thus gives more relief from pain and more certainty as to the position. He gives some very useful directions as to the application of the plaster bandage and how to prevent failures in its application. The plaster must be kept hermetically sealed to insure prompt setting. He finds that frequently the dressing does not set promptly from other causes than moisture. It may be due to the fact that the crinoline is sized with glue. He finds that glue always interferes with the setting of plaster-Paris, and suggests that by moistening the fingers and handling the material between the thumb and finger one can easily detect by its stickiness whether glue has been used in the preparation. If it has, it becomes useless for dressing. Only starch sizing will answer. The iodine test can also be resorted to. If it is impossible to get a properly sized crinoline, the goods may be washed and thoroughly rinsed in warm water. Washed bandages set quickly, but are more difficult to apply. Another obstacle seems to be the thickness of the thread and mesh work, a very coarse thread absorbing too much water, so that in squeezing it out not sufficient plaster is left in the bandage, and in the course of an hour or so the dressing becomes moist, soft and useless. The best crinoline is the brand known as "A. Virtute." Salt and alum should not be added to the water, because it renders the plaster brittle and so makes a poor support, though it hardens much more rapidly. Another objection is that it becomes more irritating to the skin, often causing excoriations which are difficult to heal. The writer's chief argument for the use of plaster seems to be the fact that it does immobilise and does maintain the parts in the desired position, thus giving certainty and, moreover, much relief of pain. He does not believe that the plaster dressing is productive of atrophy or any special tendency to anchylosis or stiffness of joints, and cites a number of cases to prove it. The easiest and, perhaps, the quickest method of removing a plaster dressing he finds is soaking the surface, which is to be cut with a large pruning knife, with hot, salt water. The paper concludes with the following summary:

Plaster-of-Paris should be dry and kept preferably in tightly sealed cans. Only the meshes of the crinoline should be filled with plaster. Crinoline sized with glue should not be used without having the glue washed out. The bandages should be moistened in lukewarm water without the addition of salt or alum. The excess of water should be removed by even pressure. All bony prominences should be well padded. At other points the dressing should be as nearly skin-fitting as possible. The bandages should be applied evenly and without tension. Each layer of plaster should be thoroughly rubbed by the hands of the operator or his assistant. These preliminaries being carried out, we reach the following conclusions as regards the advantages of plaster-of-Paris: (1) It gives comfort to the patient; (2) night cries are relieved; (3) it effectually immobilises and retains the parts in the desired position, allowing the patient to move about in bed or even walk around with ease; (4) it does not excoriate when properly applied; (5) it is questionable whether plaster alone ever produces atrophy or pathological changes of the joints; (6) it is neat and easy of application; (7) it is equally applicable to adults and children.

---

A. Vander Veer, of Albany, N. Y., (*International Journal of Surgery*, November, 1895,) makes a strong plea for a more uniform and simple classification of appendicitis, citing cases in illustration and concluding as follows:

Let me emphasise—make our classification of appendicitis as simple as possible. I would say, then, we have first, acute perforative appendicitis. Second, catarrhal appendicitis, a condition due to the bacillus communis, possibly to some traumatism, possibly to some foreign substance, relapsing in its character, one or more attacks occurring, with shorter or longer intervals; some cases accompanied with suppuration and abscess, within or without the peritoneal cavity; in some cases attacks resulting in perforation, causing death, very much as do those in the first attack of acute perforative appendicitis. The diagnosis in any event should be made as clearly and distinctly as possible, and the earlier the better. We must overcome the impression that so frequently prevails that a foreign substance causes all the trouble. The bacillus communis is the important factor. Within a period of eighteen months I have done fifteen of these operations for relapsing appendicitis without a death, and have found the appendix in all conditions possible: short, long, obliterated to a mere string, enlarged, elongated and swollen, with adhesions very extensive and embarrassing; in some cases associated with a sinus still discharging from a previous abscess; very few cases presenting a foreign substance—fecal concretion or otherwise.

---

### THE INFLUENCE OF OPERATIVE PROCEDURES IN PERITONEAL TUBERCULOSIS.

Hunter Robb (*Cleveland Medical Gazette*,) says the introduction of operative procedures in cases of peritoneal tuberculosis is interesting from a scientific as well as from a practical point of view. Even when it has been decided that operative treatment is beneficial, it is not so easy to decide in what way the good results are brought about. Stchegoleff, in the *Archives de Medicine Experimentale et d' Anatomie Pathologique* of September, 1894, gives a rèsumè of recent literature, dealing with this subject to the following effect: Peritoneal tuberculosis is met with in both adults and children. In the latter, between the ages of three and ten years, boys being more frequently attacked than the girls, except in cases accompanied by ascites, which have often been known to get well spontaneously. The introduction of operative procedures has certainly materially improved the prognosis. The first cases of abdominal section in peritoneal tuberculosis were the result of mistakes in diagnosis. In Osler's collection of ninety-six cases the abdomen was opened thirty times for supposed cysts. In the classical case of Spencer Wells, which is the first instance on record of a tubercular peritonitis cured by operation, the abdomen was opened for an ovarian cyst, whereas an encysted peritonitis was found. Koenig was the first to open the abdomen intentionally for a tubercular peritonitis, and to him belongs the credit of instituting the operation for this disease. The figures given by the different observers as to the result of the operation vary a great deal, but in general are highly satisfactory. The immediate mortality in Koenig's cases was scarcely 3 per cent. Eighty-two per cent. left the hospital in very satisfactory condition, while 65 per cent. may be said to have entirely recovered. Roersch's statistics are less satisfactory, and give only one-fourth as absolutely cured, but many materially improved. These results are questioned because the tubercle bacillus was not demonstrated in all cases. Notwithstanding, however, it is an undoubted fact that many have been cured by laparatomy, in which the tubercle bacilli were found and inoculations produced the disease in animals.

Kichensky, upon making careful experiments with guinea-pigs, found that, of the animals inoculated, those upon which laparatomy was performed survived longer than the others, although all finally succumbed to general tuberculosis. Microscopical examination showed around the tuberculous nodules a development of connective tissue having a fibrous character at the periphery and containing young elements at the center, also round epithelioid cells, and in some cases the connective tissue was so abundant that the tuberculous elements were crowded out and had disappeared.

Gatti, as a result of his experiments, held that the tubercles were resolved, and that the tuberculous peritonitis was cured without the intervention of any active inflammatory process. In an autopsy made four months after a laparatomy, Osler found upon the peritoneum hard granules surrounded by cicatricial tissue, which under the microscope appeared to be fibrous nodules containing some tubercle bacilli and giant cells. He concluded that the tubercles were undergoing a fibrous transformation and that this was the method by which cures were brought about.

Stchegoleff made experiments upon dogs, incidentally discovering that they are by no means as immune or resistant to tuberculosis as has been thought. He inoculated twenty-two dogs, of which ten were operated upon and the remaining left as "controls." All developed characteristic tubercular diseases, but those operated upon survived the "controls" a longer or shorter time, according to how soon they were operated upon. Autopsy showed that the tubercles seen on the peritoneum during the operation had undergone the peculiar fibrous change above noted, while some had entirely disappeared. The degree of retrogression of the tuberculous products varied in the different dogs, according to the time which had elapsed since the operation. The paper concludes with the following summary:

(1) Tuberculous peritonitis in dogs can be cured by laparatomy. (2) Cure is not possible unless the laparatomy is done early. (3) The retrogression of the tuberculous products is brought about principally by inflammatory reaction, which causes infiltration with embryonal cells, phagocytosis and the active development of connective tissue. The specific elements of the tuberculous process are absorbed and we have a fibrous transformation. (4) In connection with the laparatomy certain physical agents help to bring about this curative action. Among these may be reckoned the mechanical trauma which the peritoneum undergoes during the laparatomy, thermic influences, the penetration of air in the abdominal cavity, and perhaps the influence of light. By these agents an irritation is set up, and an inflammatory reaction more or less intense, which is conducive to the arrest ofthe morbid process. (5) Contrary to the opinion of Vierordt, the evacuation of the exudate is not the sole cause of the cure. In the author's cases the best results were found in those instances in which the abdomen contained no fluid. (6) Dogs must be considered as very sensitive to tuberculosis.

